# A novel PTH1R missense variant in a patient with early-onset primary hyperparathyroidism

**DOI:** 10.1016/j.bonr.2026.101948

**Published:** 2026-09-03

**Authors:** Conghui Cao, Xiaoli Wang

**Affiliations:** Department of Endocrinology and Metabolism, Institute of Endocrinology, NHC Key Laboratory of Diagnosis and Treatment of Thyroid Diseases, The First Hospital of China Medical University, Shenyang, PR China

**Keywords:** Primary hyperparathyroidism, PTH1R gene, Missense variant, Early-onset endocrine disorder

## Abstract

We report a 30-year-old male with early-onset primary hyperparathyroidism (PHPT) and incidental papillary thyroid carcinoma. Biochemical tests showed hypercalcemia, hypophosphatemia, elevated PTH and normal renal function. Planar ^99^ᵐTc-MIBI scintigraphy projected a parathyroid lesion to the left thyroid inferior pole, while surgery revealed an ectopic solitary parathyroid adenoma in the left upper mediastinum; serum calcium, phosphate and PTH normalized two months postoperatively. Whole-exome sequencing detected a novel heterozygous PTH1R variant c.982G > A (p.Gly328Ser), classified as a variant of uncertain significance by ACMG/AMP criteria. The identical variant was identified in the proband's father, whose annual routine calcium and phosphorus tests remained persistently normal, so he declined additional parathyroid-related biochemical testing. Unlike previously reported PTH1R mutations linked to skeletal/dental defects, this patient presented isolated PHPT without skeletal or dental abnormalities. Neither classic loss-of-function nor canonical gain-of-function mechanisms fully explain this unique phenotype, and no in vitro functional data are available to validate variant pathogenicity. This case expands the phenotypic spectrum of PTH1R-related disorders and offers a new genetic clue for early-onset PHPT. Further functional experiments and long-term family follow-up are needed to verify the clinical relevance of this variant.

## 1 Introduction

Primary hyperparathyroidism (PHPT) is a common endocrine disorder characterized by inappropriate parathyroid hormone (PTH) hypersecretion and hypercalcemia. Young-onset PHPT (onset age < 30 years) is highly suspected to be hereditary, and genetic testing is recommended in clinical guidelines (Newey, 2021; Bilezikian et al., 2016). Several causative genes, including *MEN1, RET, CDKN1B, CDC73, CASR, GNA11, AP2S1,* and *CCND1* have been well established in both syndromic and non-syndromic PHPT. Abnormalities in these genes contribute to the pathogenesis of sporadic and hereditary PHPT by regulating parathyroid cell proliferation, differentiation, and signaling pathways of the calcium-sensing receptor (Bilezikian et al., 2016).

The *PTH1R* gene encodes a class B G protein-coupled receptor (GPCR), which is central to the regulation of calcium-phosphate metabolism and skeletal development. Pathogenic mutations in *PTH1R* have been well-documented in conditions such as primary failure of tooth eruption (PFE), skeletal dysplasias, and pseudohypoparathyroidism. However, to our knowledge, no *PTH1R* variant has been previously associated with PHPT (Portales-Castillo et al., 2022; Zhao et al., 2023; Hendricks et al., 2017).

Herein, we present a case of early-onset PHPT in a 30-year-old Chinese patient, in whom a rare heterozygous *PTH1R* variant c.982G>A (p.Gly328Ser) was detected via whole-exome sequencing. This report describes the clinical and genetic features of the case, and discusses existing limitations and future research directions.

## 2 Case

### 2.1 Patient information and clinical presentation

A 30-year-old Chinese man was admitted due to persistent hypercalcemia and elevated PTH, which were incidentally detected during preoperative evaluation for papillary thyroid carcinoma (PTC). The patient had no personal or family history of hyperparathyroidism, other endocrine tumors, obvious bone pain, recurrent renal colic or tooth abnormalities.

### 2.2 Laboratory and imaging examinations

Laboratory examinations revealed elevated serum calcium levels (2.75 mmol/L; reference range: 2.11–2.52 mmol/L), hypophosphatemia (0.59 mmol/L; reference range: 0.85–1.51 mmol/L), and inappropriately elevated PTH levels (28.77 pmol/L; reference range: 0.66–12 pmol/L). The 24-h urinary calcium was elevated at 10.84 mmol/24 h, with a calcium-to-creatinine ratio of 0.46 mmol/mmol. Renal function indicators were normal: serum creatinine was 74 μmol/L (reference range: 57–97 μmol/L), and estimated glomerular filtration rate (eGFR) was 117.9 mL/min/1.73m^2^. The serum 25-hydroxyvitamin D concentration was 23.3 ng/mL.

Dual-phase ^99^ᵐTc-MIBI parathyroid scintigraphy showed focal radiotracer uptake at the inferior pole of the left thyroid lobe. Bone mineral density was within normal range. Abdominal ultrasound excluded nephrolithiasis.

### 2.3 Genetic testing and family analysis

**Fig. 1:**
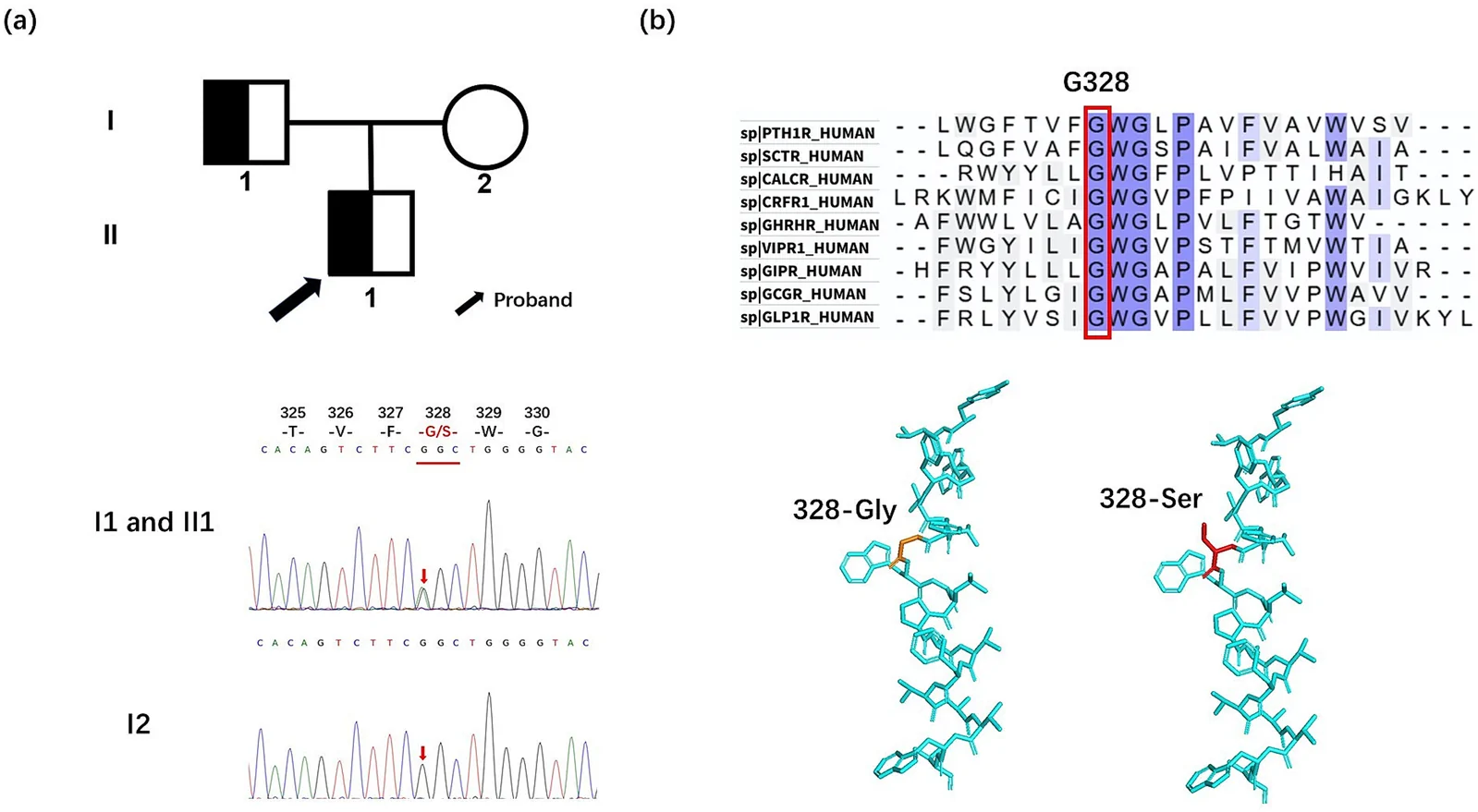
Genetic and structural characterization of the PTH1R c.982G > A (p.Gly328Ser) mutation. (a) Pedigrees of the family and sequencing chromatogram of the PTH1R mutation. A black arrow marks the proband. Males and females are indicated by squares and circles, respectively. Half-black symbols indicate individuals with confirmed PTH1R p.Gly328Ser mutation; white symbols indicate unaffected family members. (b) Multiple sequence alignment and structural schematic of p.Gly328Ser in human PTH1R (PDB ID: 7Y35) constructed using PyMOL. Amino acids surrounding the mutation site (marked with a red box) are listed. Glycine (G) at position 328 is marked with orange, and Serine (S) is marked with red.

Whole-exome sequencing identified a novel heterozygous variant *PTH1R* c.982G>A (p.Gly328Ser) in exon 10 (Fig. 1a). This variant was not found in the East Asian population of the gnomAD database. The REVEL score was 0.960, and it was classified as a variant of uncertain significance (VUS) according to ACMG guidelines (PM1, PM2, PP3).

Family segregation analysis identified the identical variant in the proband's father, while the mother had a wild-type. The Gly328 residue in the fourth transmembrane domain of PTH1R is highly conserved across GPCRs (Fig. 1b) and vital for receptor conformation and stability. The father had no history of thyroid or bone disease. He receives annual routine physical examinations with regular serum calcium and phosphorus detection, all of which showed normal levels; thus, he declined repeated serum calcium, phosphate, PTH, bone densitometry and cervical ultrasonography in this admission. After we highlighted the essential role of familial genetic verification for exploring the hereditary background of early-onset primary hyperparathyroidism, both parents consented to targeted genetic testing of this *PTH1R* variant.

### 2.4 Treatment

After diagnosis, total resection of the left thyroid lobe and thyroid isthmus was performed for papillary thyroid carcinoma, accompanied by complete excision of the ectopic parathyroid lesion identified intraoperatively in the left upper mediastinum.

Gross examination of the parathyroid specimen revealed a solitary grey-brown nodule sized 2.5 × 2.5 × 1 cm with intact capsule. Histopathological evaluation demonstrated a parathyroid tumor mainly composed of chief cells and oxyphil cells, accompanied by prominent interstitial edema. No definite vascular invasion was identified. The final pathological diagnosis was ectopic solitary parathyroid adenoma.

Serum calcium, phosphate and PTH normalized at the 2-month postoperative follow-up.

## 3 Discussion

Onset of PHPT before 30 years of age confers a high risk of hereditary causes, and current guidelines recommend routine genetic screening for such patients (Newey, 2021; Bilezikian et al., 2016). The present patient is a 30-year-old male with early-onset PHPT and an unremarkable family history, who therefore fulfilled the criteria for genetic testing. This case reinforces the value of genetic screening among young individuals presenting with PHPT.

Previous studies have linked *PTH1R* mutations primarily to parathyroid function ectopia (PFE), skeletal dysplasia and pseudohypoparathyroidism (Portales-Castillo et al., 2022; Zhao et al., 2023; Hendricks et al., 2017). To our knowledge, this is the first report of a *PTH1R* variant identified in a patient with PHPT, which broadens the phenotypic spectrum of disorders associated with *PTH1R* alterations. These findings raise the possibility that this receptor is involved in the regulation of peripheral calcium homeostasis, and that its functional impairment may potentially contribute to autonomous PTH overproduction in the parathyroid glands.

The PTH1R consists of three functional domains: the N-terminal extracellular domain (ECD) is responsible for recognizing and binding ligands; the intermediate seven-transmembrane helix domain transmits hormone-binding signals and interacts with G proteins; the C-terminal intracellular domain receives signals and subsequently interacts with G proteins to activate downstream signaling pathways (Gardella and Juppner, 2001). This receptor has a relatively large amino-terminal extracellular domain, which plays a key role in the initial binding of ligands, while the seven-helix transmembrane domain and connecting loops mediate agonist-induced receptor activation and signal transduction events (Gardella and Juppner, 2001).

**Fig. 2:**
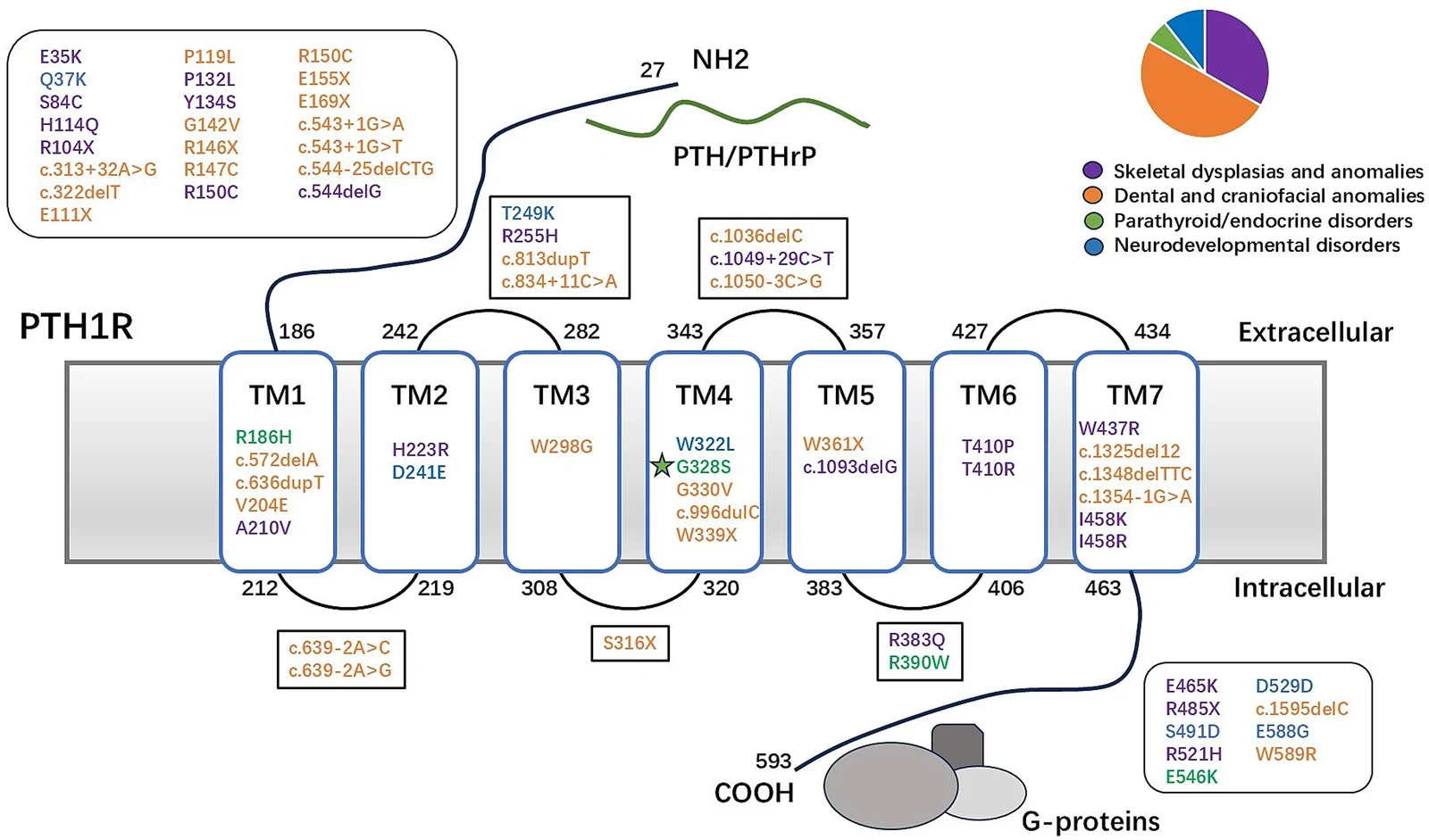
Schematic representation of the PTH1R and the distribution of previously reported pathogenic variants. The schematic depicts the topology of the PTH1R, including the N-terminal extracellular ligand-binding domain, the seven-transmembrane helices (TM1–TM7), and the C-terminal intracellular domain that couples to G-proteins. All previously reported *PTH1R* pathogenic variants associated with human diseases are mapped onto the receptor structure, with variant names labeled according to their approximate positions in the extracellular domain, transmembrane helices, and intracellular C-terminal tail. Each variant is color-coded based on its associated clinical phenotype, as summarized in the inset pie chart: purple for skeletal dysplasias and anomalies, orange for dental and craniofacial anomalies, green for parathyroid/endocrine disorders, and blue for neurodevelopmental disorders. The novel variant identified in this study, *PTH1R* p.Gly328Ser (c.982G > A), is highlighted with a green star in the fourth transmembrane helix (TM4). This figure illustrates the distribution of *PTH1R* variants across different domains and their associated phenotypes, expanding the known spectrum of PTH1R-related disorders to include primary hyperparathyroidism.

We summarized all literature on the association between *PTH1R* gene mutations and diseases (from HGMD) and found that diseases caused by this gene mutation can currently be divided into 4 categories (Fig. 2). Among them, the majority (∼85%) can lead to skeletal and dental abnormalities (Portales-Castillo et al., 2022; Hendricks et al., 2017), mainly caused by loss-of-function mutations (frameshift, splicing, and nonsense mutations). In contrast, few point mutations (∼15%) are associated with neurodevelopmental disorders (7 cases) and endocrine/parathyroid diseases (3 cases). For example, the homozygous mutation p.Arg186His located in the first transmembrane helix can cause pseudohypoparathyroidism type 1b (PHP1B), and its mechanism is mainly characterized by selective impairment of the receptor's ability to recognize and bind parathyroid hormone (PTH) ligands, rather than a significant decrease in receptor expression level (Portales-Castillo et al., 2022; Guerreiro et al., 2016). In a study related to *GCM2* gene variants, another patient with hypoparathyroidism also carried the *PTH1R* p.Arg390Trp variant; however, this variant is located in the cytoplasmic region and does not involve key domains for ligand binding or signal transduction, so its correlation with the hypoparathyroidism phenotype is unclear (Garcia-Castano et al., 2021). The *PTH1R* p.E546K variant has been reported to be associated with familial gastric neuroendocrine tumors; this variant is located in the intracellular C-terminal region of the receptor and acts synergistically with *ATP4A* gene mutations, leading to gastric acid secretion disorders and hypergastrinemia, and may also be associated with the patient's concurrent hypothyroidism and rheumatoid arthritis (Calvete et al., 2017).

The proband harbors a novel missense variant within the fourth transmembrane domain of PTH1R, which locates at the cytoplasmic terminus of the transmembrane helix, a region where several previously reported PTH1R activating variants have also been identified. Three well-characterized activating PTH1R mutations are linked to Jansen metaphyseal chondrodysplasia, a rare dwarfism syndrome accompanied by hypercalcemia (Obiezu et al., 1993). These variants (p.Arg223His, p.Thr410Pro, p.Ile458Arg) are located at the cytoplasmic ends of the second, sixth and seventh transmembrane domains, respectively. Functional in vitro studies have confirmed that these mutations trigger ligand-independent constitutive activation of the cAMP signaling pathway (Obiezu et al., 1993; Calvi and Schipani, 2000). A notable clinical difference is observed between the two conditions. Patients with Jansen metaphyseal chondrodysplasia generally present with suppressed endogenous PTH, whereas our patient showed typical biochemical features of primary hyperparathyroidism. Although constitutive PTH1R activation leads to hypercalcemia in Jansen metaphyseal chondrodysplasia, this mechanism cannot fully explain the pathophysiological changes seen in PHPT.

After reviewing all reported PTH1R pathogenic variants and relevant functional studies summarized in Fig. 2, we reasoned that p.Gly328Ser is unlikely to act as a loss-of-function mutation. Severe skeletal and dental malformations are typical hallmarks of loss-of-function PTH1R variants such as frameshift and splicing mutations, while isolated missense variants tend to produce mild phenotypic perturbations. Nevertheless, canonical gain-of-function PTH1R mutations also fail to fully explain our patient's biochemical manifestations: although constitutive receptor activation in peripheral organs accounts for hypercalcemia, elevated calcium would normally suppress parathyroid PTH synthesis and secretion via negative feedback, which conflicts with the autonomous PTH hypersecretion and solitary parathyroid adenoma detected in this subject.

In the absence of cellular functional evidence, we can only propose tentative mechanistic speculations to reconcile this unique phenotype. This transmembrane substitution may trigger tissue-specific divergent signaling effects, conferring weak constitutive activity in bone and kidney to raise circulating calcium while interfering with intracellular calcium-sensing feedback in parathyroid cells to break physiological suppression of PTH production. Furthermore, this single amino acid alteration only mildly disturbs the conformation of the fourth transmembrane helix, resulting in limited functional impairment insufficient to trigger overt skeletal or dental lesions that predominate in most documented PTH1R mutation carriers. It is also plausible that unidentified germline or somatic genetic modifiers synergize with this PTH1R variant to promote parathyroid adenoma development. Further in vitro functional characterization is essential to verify these conjectures and clarify the exact pathogenic pathway of this novel variant.

Further in vitro functional characterization is essential to verify these conjectures and clarify the exact pathogenic pathway of this novel variant. The seven transmembrane α-helices (TM1-TM7) of GPCRs are their structural backbone and functional core. The fourth transmembrane domain of Class B GPCRs was found to be a highly conserved characteristic motif. For example, glycine (G328) is a core conserved residue of TM4; its minimal volume provides the necessary space for the tight packing of transmembrane helices and is crucial for maintaining receptor conformational stability. After G328 is mutated to Ser (S) with a polar side chain, the structure of TM4 changes in three dimensions: steric hindrance, disruption of the hydrophobic environment, and reduction of helix flexibility, which may further affect the conformational stability and signal transduction function of PTH1R.

The prevalence of concomitant PTC in PHPT ranges from 2% to 15% (Jeong et al., 2020). Currently, whether the coexistence of PTC and PHPT is accidental or associated remains controversial, which may be related to factors such as elevated PTH, elevated blood calcium, and decreased vitamin D^12^. No genetic abnormalities of multiple endocrine neoplasia, such as *MEN1* and *CDKN1B*, were found in this patient. To date, there are no reports on the association between PTH1R and thyroid cancer.

Several limitations of the current study should be acknowledged. First, we failed to obtain real-time serum PTH, calcium and phosphorus measurements from the proband's father during this hospitalization. Although his annual physical examinations revealed persistently normal serum calcium and phosphate levels, the lack of simultaneous PTH data limits comprehensive genotype-phenotype correlation analysis within this pedigree. Second, this report only describes a single clinical case without supporting in vitro functional verification of the *PTH1R* c.982G>A (p.Gly328Ser) variant, so we cannot draw definitive conclusions regarding its pathogenic role in PHPT.

In summary, we identified a novel heterozygous *PTH1R* missense variant in a young patient with early-onset primary hyperparathyroidism complicated by papillary thyroid carcinoma. This case expands the known phenotypic spectrum of diseases associated with *PTH1R* gene alterations. Further cellular functional experiments and long-term clinical follow-up of this pedigree are warranted to clarify the causal link between this variant and autonomous parathyroid hormone overproduction.

## CRediT authorship contribution statement

**Conghui Cao:** Writing – original draft, Investigation. **Xiaoli Wang:** Writing – review & editing, Writing – original draft, Supervision, Investigation.

## Declaration of Generative AI and AI-assisted technologies in the writing process

During the preparation of this manuscript, the authors used Doubao for English language polishing. All AI-generated content was carefully reviewed and revised by the authors. The authors take full responsibility for the entire content of this published article.

## Declaration of competing interest

The authors declare that the research was conducted in the absence of any commercial or financial relationships that could be construed as a potential conflict of interest.

## Data Availability

Data will be made available on request.
